# Influence of short-term hypoxia exposure on dynamic visual acuity

**DOI:** 10.3389/fnins.2024.1428987

**Published:** 2024-07-10

**Authors:** Yuchen Wang, Jiaxing Xie, Xinli Yu, Yihe Liu, Zesong Wang, Anqi Guo, Yi Ding, Xinzuo Zhou, Siru Liu, Jiaxi Li, Chengkai Zhou, Yuanhong Li, Ziyuan Liu, Xuemin Li, Li Ding

**Affiliations:** ^1^Department of Ophthalmology, Peking University Third Hospital, Beijing, China; ^2^Beijing Key Laboratory of Restoration of Damaged Ocular Nerve, Peking University Third Hospital, Beijing, China; ^3^School of Biological Science and Medical Engineering, Beihang University, Beijing, China

**Keywords:** dynamic visual acuity, simulated high-altitude environment, angular velocity, hypoxia, visual function

## Abstract

**Background:**

To quantify the changes in dynamic visual acuity (DVA) and explain the hidden reasons after acute exposure to hypobaric hypoxia status.

**Methods:**

The study group comprised 18 healthy male and 15 healthy female participants aged 20–24 years old. DVA was measured with the self-developed software of Meidixin (Tianjin) Co., Ltd. Measurements were taken at eight altitudes. Data analysis was performed using the Kolmogorov–Smirnov test, paired sample *T*-test, and two-way repeated measures analysis of variance (ANOVA) for repeated measurements.

**Results:**

At constant altitude, DVA showed an overall decreasing trend with increasing angular velocity and a fluctuating decrease at the vast majority of altitudes. At constant angular velocities, DVA gradually increased with altitude, with the most pronounced increase in DVA at altitude 5, and thereafter a gradual decrease in DVA as altitude increased. Finally, as altitude decreased, DVA increased again and reached a higher level at the end of the experiment, which was superior to the DVA in the initial state.

**Conclusion:**

Under a hypobaric hypoxic environment at high altitude, DVA was affected by the angular velocity and the degree of hypoxia, manifesting as an increase or decrease in DVA, which affects the pilot's observation of the display and control interfaces during the driving process, acquisition of information, and decision-making ability, which in turn may potentially jeopardize the safety of the flight.

## 1 Introduction

In recent years, the field of high-altitude has become increasingly important in the context of human survival. However, the lack of adaptation to the hypobaric hypoxia of a high-altitude environment presents significant challenges for human beings, particularly in relation to changes in important functions such as visual information during the flight process. A decline in the function of human organs may result in the failure of the information reception and decision-making systems, which could have irreversible and dangerous consequences. Currently, the rapid advancement of aerospace technology has enabled the formation of a closed, pressurized space in a multitude of aircraft types. However, there persists a certain degree of oxygen partial pressure reduction and other challenges. Certain classes of aircraft, including helicopters, do not typically carry oxygen supply equipment, which contributes to the heightened risk of pilot hypoxia. Existing studies have demonstrated that the eye is one of the most sensitive organs to changes in oxygen concentration (Akberova et al., 2016). Hypoxia will therefore cause changes in the structure and function of the eye, including the cornea (Pang et al., 2021), pupil (Krusche et al., 2020), lens (Brennan et al., 2020), vitreous body (dell'Omo et al., 2013), and retina (Shinojima et al., 2021), which in turn will cause changes in visual function, including static visual acuity and dynamic visual acuity (DVA).

Previous studies have demonstrated that the refractive state of the human eye is altered in high-altitude environments. This phenomenon was exemplified by the study of Gekeler et al. (2019). Although a statistically significant decrease in static visual acuity was not observed, the static visual acuity of the human eye at high-altitude environments (4,559 m) was higher than that of the human eye when it was located at sea level one day earlier (LogMAR), suggesting that hypobaric hypoxia leads to differences in the refractive state of the human eye, which will undoubtedly affect the observation of moving objects. Krusche et al. (2020) conducted further investigations into the changes in DVA of 11 trekkers at different altitudes (154 m, 3,647 m, and 4,554 m) and motion contrasts (100%, 50%, 30%, and 20%). In their observations, which spanned 8 days, the researchers that DVA at 100% motion contrasts increased with elevated altitude. Furthermore, they observed that DVA began to decrease after reaching 4,554 meters. Furthermore, at 30% and 20% motion contrast, an increase in altitude was associated with a decline in DVA. In this context, motion contrast is defined as the percentage of moving pixels in the Landolt ring relative to stationary pixels in the background (Wist et al., 1998; Gekeler et al., 2019). The results demonstrated that DVA is significantly influenced by hypoxia. However, the aforementioned study was conducted under chronic hypobaric hypoxic conditions, which cannot fully represent the instantaneous changes in DVA that occure after acute exposure to a hypobaric hypoxic environment.

The objective of this study is to further quantify the trends of DVA under acute exposure to hypobaric hypoxia and to analyze the reasons for them. This will help to clarify the effect of a hypobaric hypoxic environment at high altitude on the visual function of the human eye. This will also help to further simulate the visual obstacles that can be caused by the emergence of acute hypoxia in the course of flight. This will provide a reference for instrument panels in aircraft safety design.

## 2 Methods

### 2.1 Subjects

The prospective study enrolled 33 healthy, physically fit volunteers, aged 20–24 years old, with 18 males and 15 females. All subjects were of Han descent and resided at altitudes between 40 and 250 meters above sea level. None of the volunteers in this study had any systemic or diseases affecting the visual axis (e.g., glaucoma, shallow anterior chamber, narrow atrial angle, etc.) or a history of ophthalmic surgery, intraocular lenses, or contact lenses. Subjects were required to ensure that they had sufficient rest prior to the test and to refrain from consuming caffeinated beverages, such as coffee or tea, in the hours leading up to the experiment. The tenets of the Declaration of Helsinki were adhered to throughout the research process, and the study was approved by the Biomedical Ethics Committee of Beihang University. Prior to the commencement of the study, each participant was provided with a comprehensive explanation of the nature and potential consequences of the research. This was followed by the acquisition of written informed consent.

### 2.2 Ascent profile and measurements

**Figure 1:**
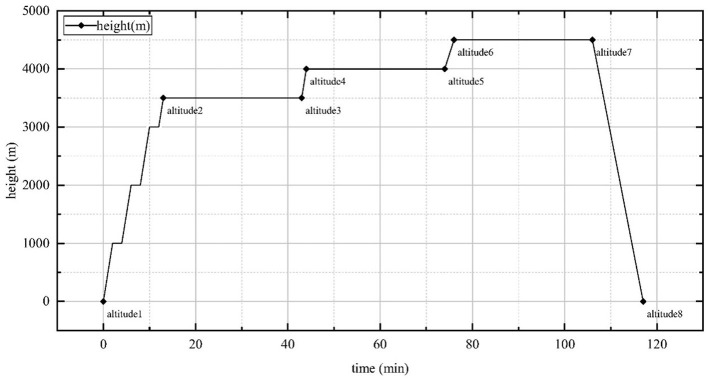
Highland complex environment simulation chamber altitude rise and fall time points, lasting a total of 2 h. Altitude 1: ground; Altitude 2: 3,500 m; Altitude 3: 3,500 m, 30min; Altitude 4: 4,000 m; Altitude 5: 4,000 m, 30min; Altitude 6: 4,500 m; Altitude 7: 4,500 m, 30 min; Altitude 8: end of experiment.

The experiment was conducted in a high-altitude complex environment simulation chamber at the BHU High-Precision Medicine Center under the supervision of a professional physician throughout the study. The temperature and humidity within the chamber were maintained at a range of 23.3 to 26.5°C and 30–35%, respectively. The experimental chamber ascended at a speed of 10 m/s, and 2 minutes of habituation were included after every 1,000 m. When the altitude of the simulation chamber reached 3,500 m, 4,000 m, and 4,500 m above sea level, the subjects completed their adaptation to high-altitude hypoxia with 30 min of habituation, and then the simulation chamber descended at a speed of 7 m/s. The DVA of the subjects was quantified prior to ascent, at arrival, and following habituation at 3,500 m, 4,000 m, and 4,500 m above sea level, and at the conclusion of the experiment. The specific experimental altitude changes are depicted in Figure 1.

The BCVA was recorded as the logarithm of the minimum angle of resolution (LogMAR). IOP was measured in both eyes with a portable handheld iCare tonometer, which has a disposable test bar (approximately 0.9 mm in diameter, with a contact area of approximately 0.65 mm^2^) and measures IOP in mmHg by making ejection contact with the apex of the corneal surface. Refraction was measured by a portable handheld refractometer (Topcon, Japan) in diopters (D). The refractometer was based on the principle of the Hartmann–Shack wavefront sensor for the detection of ocular refraction and can be used for initial screening and determination of refraction in the human eye.

The self-developed dynamic vision software of Meidixin (Tianjin) Co., Ltd. was utilized to quantify DVA. All tests were conducted in a natural brightness environment during the daytime. The direction of motion of the Snellen reticle was from right to left, and the opening direction of the reticle was up, down, left, or right, appearing in a randomized order. The measurements were taken for each subject from an angular velocity of 20 s-1, with the LogMAR value starting from 1.0 during the test and being adjusted downward when the subject correctly responded to the opening direction of the reticle four times. The aforementioned measurements were repeated until the lowest possible LogMAR value was identified. Once the angular velocity of 20 s-1 had been completed, the same procedure was repeated for the 40 s-1, 60 s-1, and 80 s-1 conditions. The measurement method was maintained throughout the experiment, with all heights being treated consistently.

### 2.3 Statistical analysis

A Kolmogorov–Smirnov test was conducted to ascertain the presence of a normal distribution. The paired sample *T*-test was employed to assess the disparities between four angular velocity points at a constant altitude and between eight altitude points at a constant angular velocity in terms of the LogMAR value. A two-way repeated measures analysis of variance (ANOVA) was employed to ascertain the impact of alterations in angular velocity at varying altitudes on the LogMAR value of the subjects. A *p* < 0.05 was considered statistically significant. All statistical analyses were conducted using a commercially available software package (SPSS for Windows, v. 28.0, IBM).

## 3 Results

### 3.1 Participant general demographics

A total of 33 participants were enrolled in our study. Their age ranged from 20 to 24 years. The BCVA was 0.11 ± 0.13 and 0.10 ± 0.11 in the right eye and left eye, respectively. All subjects safely reached the simulated altitude of 4,500 m and completed the experiment safely without oxygen supplementation. The demographic characteristics of the participants are summarized in Tables 1, 2.

**Table 1:** Patient general demographics (*N* = 33).

**Characteristic**	**Value**
Gender (male/female)	18/15
Age, years, mean ± SD	21.2 ± 1.2
**BCVA, LogMAR**	
Right eye, mean ± SD	0.11 ± 0.13
Left eye, mean ± SD	0.10 ± 0.11
**IOP, mm Hg**	
Right eye, mean ± SD	15.5 ± 2.7
Left eye, mean ± SD	15.4 ± 2.7
**Refraction**	
**SPH**	
Right eye, mean ± SD	−5.31 ± 3.33
Left eye, mean ± SD	−4.92 ± 3.33
**CYL**	
Right eye, mean ± SD	−0.91 ± 0.87
Left eye, mean ± SD	−0.89 ± 0.93

BCVA, best correct vision acuity; IOP, intraocular pressure; SD, standard deviation; SPH, spherical lens; CYL, cylinder.

**Table 2:** Blood Oxygen Saturation at different altitudes.

**Altitude**	**Blood oxygen saturation (%)**
Ground	98.33 ± 0.85
3,500 m	87.97 ± 1.90
3,500 m, 30 min	84.76 ± 1.75
4,000 m	81.85 ± 1.70
4,000 m, 30 min	80.21 ± 2.37
4,500 m	75.70 ± 1.49
4,500 m, 30 min	74.30 ± 1.93
End of experiment	92.21 ± 2.07

Data are expressed as mean ± standard deviation.

### 3.2 Variation in the LogMAR value with angular velocity at constant altitude

**Figure 2:**
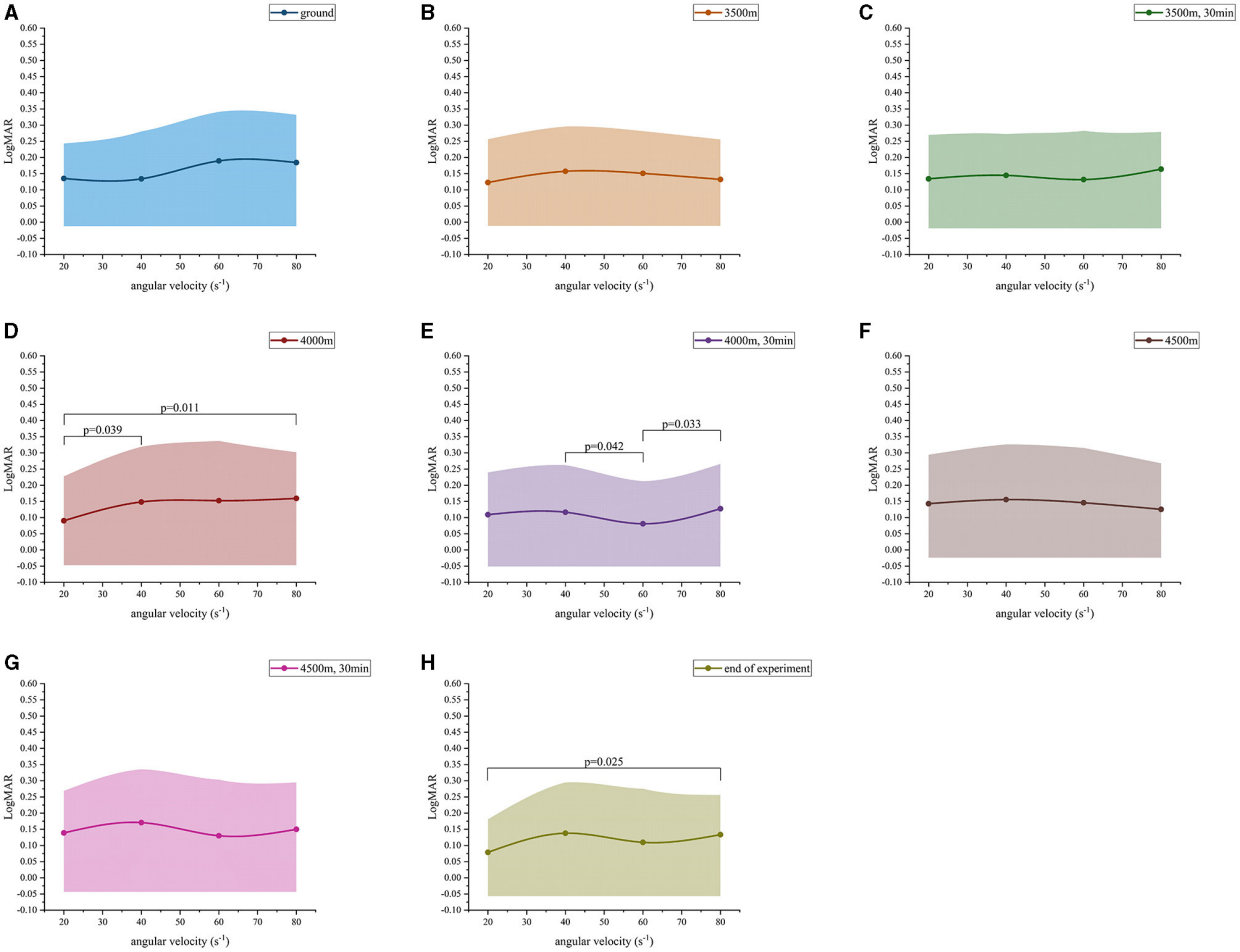
Dynamic visual acuity (LogMAR) variation values at different angular velocities. **(A)** Ground; **(B)** 3,500 m; **(C)** 3,500 m, 30 min; **(D)** 4,000 m; **(E)** 4,000 m, 30 min; **(F)** 4,500 m; **(G)** 4,500 m, 30 min; **(H)** end of experiment.

At altitudes 1, altitude 2, altitude 3, altitude 6 and altitude 7, the LogMAR values demonstrated a fluctuating upward trend as the angular velocity increased (Figures 2A–C, F, G) (Table 3). However, no significant differences were observed in the LogMAR values at each angular velocity. Additionally, the LogMAR values demonstrated a fluctuating upward trend with increasing angular velocity at altitudes 5 and 8 (Figures 2E, H). At altitude 5, the LogMAR value at an angular velocity of 60 s^−1^ (0.08 ± 0.13) was notably smaller than the LogMAR value at an angular velocity of 40 s^−1^ (0.12 ± 0.15, *p* = 0.042) and 80 s^−1^ (0.13 ± 0.14, *p* = 0.033). At altitude 8, the LogMAR value at an angular velocity of 20 s^−1^ (0.08 ± 0.10) was found to be significantly lower than the LogMAR value at an angular velocity of 80 s^−1^ (0.13 ± 0.12, *p* = 0.015). In contrast to the fluctuating upward trend observed at the preceding even altitudes, at altitude 4 **(**Figure 2D), the LogMAR value exhibited a gradual increase with increasing angular velocity, reaching its highest level at an angular velocity of 80 s^−1^. The LogMAR value at an angular velocity of 20 s^−1^ (0.09 ± 0.14) was notably smaller than the LogMAR value at an angular velocity of 40 s^−1^ (0.15 ± 0.17, p = 0.039) and 80 s^−1^ (0.16 ± 0.14, *p* = 0.011). In summary, when the height was maintained, DVA demonstrated a general downward trend with increasing angular velocity.

**Table 3:** Dynamic visual acuity (LogMAR) at different altitudes and angular velocities.

**Altitude**	**Angular velocity = 20 s^−1^**	**Angular velocity = 40 s^−1^**	**Angular velocity = 60 s^−1^**	**Angular velocity = 80 s^−1^**
Ground	0.14 ± 0.11	0.13 ± 0.15	0.19 ± 0.15	0.18 ± 0.15
3,500 m	0.12 ± 0.13	0.16 ± 0.14	0.15 ± 0.13	0.13 ± 0.12
3,500 m, 30 min	0.13 ± 0.14	0.14 ± 0.13	0.13 ± 0.15	0.16 ± 0.12
4,000 m	0.09 ± 0.14	0.15 ± 0.17	0.15 ± 0.18	0.16 ± 0.14
4,000 m, 30 min	0.11 ± 0.13	0.12 ± 0.15	0.08 ± 0.13	0.13 ± 0.14
4,500 m	0.14 ± 0.15	0.16 ± 0.17	0.15 ± 0.17	0.13 ± 0.14
4,500 m, 30 min	0.14 ± 0.13	0.17 ± 0.16	0.13 ± 0.17	0.15 ± 0.15
end of experiment	0.08 ± 0.10	0.14 ± 0.16	0.11 ± 0.17	0.13 ± 0.12

Data are expressed as mean ± standard deviation.

### 3.3 Variation in the LogMAR value with altitude at constant angular velocity

**Figure 3:**
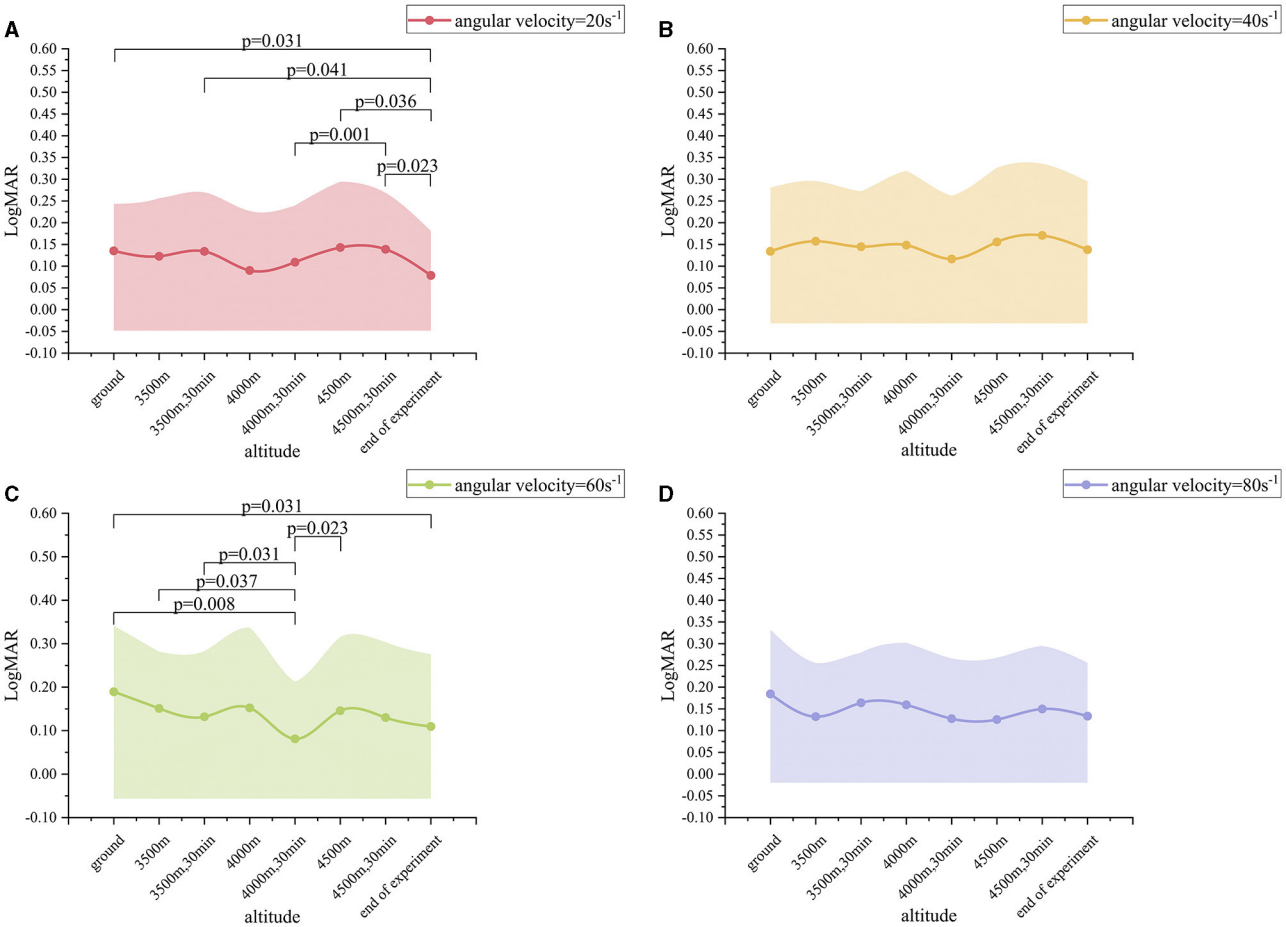
Dynamic visual acuity (LogMAR) variation values at different altitudes. **(A)** Angular velocity = 20 s^−1^; **(B)** Angular velocity = 40 s^−1^; **(C)** Angular velocity = 60 s^−1^; **(D)** Angular velocity = 80 s^−1^.

When the angular velocity was 20 s^−1^, the LogMAR value exhibited a change with altitude. From altitude 1 to altitude 5, the LogMAR value gradually decreased from 0.14 ± 0.11 to 0.11 ± 0.13. However, after the altitude was raised to altitude 7, the LogMAR value gradually rose to 0.14 ± 0.13. At altitude 8, the LogMAR value was then 0.08 ± 0.10, representing a decrease from the values observed at the other seven altitudes (Figure 3A). In particular, the LogMAR value at altitude 8 was notably lower than that at altitude 1, altitude 3, altitude 6, and altitude 7 (p-value of 0.031, 0.041, 0.036, and 0.023, respectively). Additionally, the LogMAR value at altitude 5 was significantly lower than that at altitude 7 (*p* = 0.001). When the angular velocity was 60 s^−1^, the changes in the LogMAR value were found to be slightly different from those observed at 20 s^−1^. From altitude 1 to altitude 5, the LogMAR value exhibited a gradua decreased from 0.19 ± 0.15 to 0.08 ± 0.13, but then gradually rose to 0.13 ± 0.17 after the altitude was raised to altitude 7. At altitude 5, the LogMAR value was then 0.08 ± 0.13, which was lower than the LogMAR value at all of the other seven altitudes (Figure 3C). In particular, the LogMAR value at altitude 5 was remarkably less than that at altitude 1, altitude 2, altitude 3, and altitude 6 (*p*-value of 0.008, 0.037, 0.031, and 0.023, respectively). Additionally, the LogMAR value at altitude 8 was considerably lower than that at altitude 1 (p = 0.031). When the angular velocity was 40 s^−1^ and 80 s^−1^, the changes in the LogMAR value were comparable to those observed at 60 s-1: from altitude 1 to altitude 5, the LogMAR value exhibited a gradual decrease, but a gradual increase after the altitude was raised to altitude 7. At altitude 5, the LogMAR value was found to be lower than that observed at any of the other seven altitudes (Figures 3B, D). However, no significant differences were observed when comparing the two-by-two data from altitudes 1 to 8 when the angular velocity was 40 s-1 and 80 s-1. In conclusion, when angular velocity was held constant, the exhibited a gradual improvement with increasing altitude, reaching a maximum at altitude 5, while it gradually declined at altitudes 6 and 7, and then rebounded to a higher level again at altitude 8.

### 3.4 The effect of varying angular velocity at different altitudes on the LogMAR value

A two-way repeated measures ANOVA was employed to ascertain the impact of alternations in angular velocity at varying altitudes on the LogMAR value of the subjects (Table 4). For the interaction item angular velocity^*^ altitude, the variance-covariance matrices of the dependent variables were found to be unequal, as determined by Mauchly's spherical hypothesis test, χ^2^(230) = 574.672, *p* < 0.001, corrected by the Greenhouse-Geisser method ε = 0.420. The interaction between angular velocity and altitude had no statistically significant effect on the LogMAR value, as indicated by the F-value (8.823, 282.323) = 1.138 and *P*-value (0.336). Consequently, it was necessary to interpret the main effects of the two within-subject factors (angular velocity and altitude). The variance-covariance matrices of the dependent variables differed in their equality as determined by Mauchly's spherical hypothesis test, χ^2^(5) = 11.515, p = 0.042, corrected by the Huynh-Feldt method ε=0.918. The main effect of angular velocity on the LogMAR value was statistically significant, with F (2.754, 88.114) = 2.873 and p = 0.045. For altitude, the variance-covariance matrices of the dependent variables were equal as determined by Mauchly's spherical hypothesis test, χ^2^(27) = 39.166, p = 0.063. The main effect of altitude on the LogMAR value was statistically significant, with F (7, 224) = 2.345 and p = 0.025.

**Table 4:** Two-way repeated measures ANOVA results.

**Factors**	**Wilks' Lambda value**	**F value**	***P* value**
Angular velocity	0.815	2.873	0.045
Altitude	0.638	2.345	0.025
Angular velocity^*^altitude	0.234	1.138	0.336

## 4 Discussion

DVA, or dynamic visual acuity, refers to the ability to capture, decompose, and perceive images of moving objects while observing (Jiang et al., 2021). For high-altitude flight, pilots must constantly observe the display and control interfaces and the external environment in a relatively non-stationary state in order to rapidly and accurately obtain real-time information related to flight safety, which is crucial to ensure the stable operation of the aircraft. This necessitates that the pilot maintains optimal visual performance during flight. In this study, the software developed by Medixin (Tianjin) Co., Ltd. was utilized to investigate the DVA following acute exposure to a simulated low-pressure anoxic environment.

In this study, it was observed that, with the exception of 4,000 m (altitude 4), the DVA of the other seven altitudes exhibited fluctuations and a decline with an increase in angular velocity. Consequently, the faster the Snellen reticle, the more pronounced the DVA. This finding is consistent with previous research by Ishigaki and Miyao (1993), who tested an angular velocities of 300 s^−1^ and found that the angle of each opening angle of the Landolt ring (8°, 14°, 28°, and 42°), and the angle of the Landolt ring were significantly elevated. When subjects were able to distinguish the Landolt rings accurately, angular velocity decreased compared with the initial angular velocity, indicating that an increase in angular velocity would also increase the difficulty of accurately judging the opening direction of the Landolt ring. The aforementioned trend of changes was not affected by high-altitude hypobaric hypoxia in our study. Although hypoxia has been shown to cause sympathetic excitation (Panjwani et al., 2006) and changes in pupil diameter (Stepanek et al., 2014), the present study indicates that DVA remains negatively correlated with angular velocity.

It is of note that at each angular velocity, DVA at 3,500 m, 4,000 m, and 4,500 m all exhibited a reduction in comparison to DVA at the end of the experiment (altitude 8) (Phenomenon 1). In other words, hypoxia can result in reduction in DVA in the human eye. In a study by Connolly and Hosking (2009), the effects of mild hypoxia (14.1% oxygen concentration), normoxic air and hyperoxygenation (100% oxygen concentration) on mesoscopic sensitivity were compared. The results indicated that, when the background brightness is low (1 cd/m^2^), mesoscopic sensitivity decreased with increasing hypoxia levels. This finding is consistent with the results of our study. In 2020, Krusche et al. (2020) conducted a study investigating the DVA of healthy individuals at altitudes of 3,647 m and 4,554 m. The results demonstrated that DVA exhibited a gradual decrease with increasing altitude at 20% and 30% motion contrast. Conversely, at 20% motion contrast, the DVA exhibited a gradual decrease with increasing altitude. The DVA above the macular center was found to be significantly reduced.

Furthermore, hypoxia was classified according to altitude (Davis and Hackett, 2017): moderate altitude (mild hypoxia) within 1,520–2,440 m, high altitude (moderate hypoxia) within 2,440–4,270 m, and ultra-high altitude (severe hypoxia) within 4,270–5,490 m. In our study, different levels of hypoxia resulted in variations in DVA (phenomenon 2). For instance, at all angular velocities, the DVA results were inferior at mild hypoxia (3,500 m and after 30 min of habituation) in comparison to moderate hypoxia (4,000 m and after 30 min of habituation). when angular velocity is 60 s^−1^, the DVA at altitude 3 (3,500 m and after 30 min of habituation) was significantly lower than that at altitude 5 (4,000 m and after 30 min of habituation). Schatz et al. (2013) postulated that oxidative stress induced by altitudes above 4,500 m would result in more prolonged hypoxia-relate damage to the central area of the inner layer of retina. Furthermore, we conducted a habituation period at altitudes of 3,500 m, 4,000 m, 4,500 m for 30 min upon reaching the specified altitude. Our findings indicated that sustained hypoxia resulted in a further decline in DVA (Phenomenon 3). When angular velocities were 20 s-1 and 80 s-1, DVA decreased at 3500 m (altitude 3) after 30 min of acclimation compared to 3,500 m (altitude 2), although there was no significant difference. When the angular velocity is 20 s-1, DVA decreases after 30 min of adaptation at 4,000 m (altitude 5) compared to 4,000 m (altitude 4). When the angular velocities were 40 s-1 and 80 s-1, DVA decreased after 30 min of adaptation at 4,500 m (altitude 7) compared to 4,500 m (altitude 6). Benedek et al. (2002) monitored the visual contrast sensitivity of 14 healthy males at a simulated altitude of 4,500 m. They found that with the extension of hypoxia time (0 min, 5 min, and 10 min), the blood oxygen saturation of the subjects gradually decreased. The dynamic visual contrast sensitivity of the subjects exhibited a decline with the extension of the hypoxia time. Nevertheless, due to the limited monitoring period, no significant differences were observed.

In light of the above phenomena, it is appropriate to initiate a discussion on the following points: Phenomenon 1 suggests that the reduction in DVA resulting from hypoxia may be attributed to the impact of retinal hypoxia and ischemia on the conduction of visual pathways. A review of previous literature reveals that the M pathway in the visual pathway (Connolly, 2011) is primarily associated with the transmission of motor visual information, which involves the participation of multiple brain regions. To illustrate, the M retinal ganglion cells receive information and transmit it to the M cell layer on the ventral side of the lateral geniculate nucleus (Galletti and Fattori, 2018; VerMaas et al., 2019). Subsequently, it activates the V1, V2, and V5 regions of the primary visual cortex, primarily the motor visual cortex, which plays an important role in motor vision transformation. Schatz et al. (2013) postulated that retinal cells, including ganglion cells, cones, and rods, are unable to maintain normal physiological functions under hypoxic conditions. Furthermore, the inner layer of the retina exhibits a greater tolerance to hypoxia than the outer layer, as observed by electroretinogram (ERG). Short periods of hypoxia (5 min) have been observed to affect the depolarization and hyperpolarization of retinal bipolar cells (Feigl et al., 2007). Concurrently, hypoxia results in a reduction in the metabolic status of pyramidal and rod cells, which is evidenced by the accumulation of lactic acid through anaerobic glycolysis and a decline in cellular activity. Consequently, this will further affect the transmission of electrophysiological signals (Nassi and Callaway, 2009; Ramsey and Arden, 2015), thus affecting the transmission efficiency of the M pathway, resulting in a decrease in DVA under hypoxic conditions. Phenomenon 2 indicates that the decline in DVA during mild hypoxia is considerably less pronounced than that observed during moderate hypoxia. It is hypothesized that this phenomenon may be attributed to the fact that the human eye has not yet adapted to acute hypoxia. Following the initial experience of mild hypoxia, retinal cells respond rapidly to hypoxia in a compensatory manner, while ganglion cells and other inner retinal cells gradually tolerate hypoxia and undergo a slow repair process. This results in a gradual increase in DVA. In the case of severe hypoxia (4,500 m, after 30 min of adaptation), the decrease in DVA of the human eye was most significant when compared with altitude 8, regardless of angular velocity. The results of the ERG test indicate that paracrine cells, bipolar cells, and interplexus cells, which are responsible for conducting neurotransmitters, exhibit more pronounced degeneration with the prolongation of hypoxia time (Schatz et al., 2013). Consequently, the inner layer of retinal cells is affected by prolonged hypoxia, resulting in a notable reduction in hypoxia tolerance, which has a more pronounced impact on DVA.

Finally, at the conclusion of the experiment, our DVA at this point (altitude 8) has demonstrably improved in comparison to the initial state of the ground (altitude 1). It was postulated that this phenomenon may be attributed to the fact that retinal blood flow increases as blood vessels dilate and circulation returns during the rapid transition from severe hypoxia to non-hypoxia, while the brain is abnormally sensitive to reperfusion (Rosengarten and Kaps, 2010). Cells associated with the M pathway, such as ganglion cells in the inner layer of the retina, receive an adequate supply of oxygen, which enables them to compensate for the lack of oxygen and become more sensitive to signals of various wavelengths. This results in an increase in the strength of the input signal and the transmission rate of visual information. Furthermore, the normal functioning of the primary visual cortex is contingent upon an adequate blood supply within the brain. The increase in aerobic glycolysis provides sufficient energy for the visual cortex. Consequently, it can be postulated that a rapid increase in blood oxygen levels results in an enhancement of electrophysiological cell-cell signal transduction, thereby further augmenting DVA.

Nevertheless, the experiment was not without limitations. One such limitation was the familiarity of the subjects with the dynamic vision detection software, despite the provision of DVA training prior to the experiment. In order to reduce the experimental error caused by the participant factor, it is recommended that the experiment be repeated at each height. Nevertheless, prolonged exposure to high-altitude environments with low air pressure and low oxygen can result in adverse effects on bodily functions. To ensure the safety of the participants, we were constrained to perform a single measurement task at each altitude, which inevitably resulted in a lack of reliability in our findings. In the future, we will continue to repeat DVA measurements under low pressure, low oxygen, and high-altitude conditions, and complete electrophysiological examinations, including ERG, to further clarify the causes of DVA changes caused by low pressure and low oxygen and explore the related mechanisms. Consequently, the objective is to elucidate the underlying mechanisms of DVA alterations in order to anticipate potential visual dysfunction during emergency situations that pilots may encounter during flight and to propose solutions to these emergencies.

## 5 Conclusion

In conclusion, this study aimed to investigate the effects of acute hypobaric hypoxia on DVA, which builds upon previous research that solely examined the impact of chronic hypobaric hypoxia on vision. The data presented in this study provide the first comprehensive analysis of DVA at different altitudes and angular velocities. In the future, we intend to further investigate the distinctions and functions of enriched genes in the M and P pathways in hypobaric hypoxia environments, as well as the physiological effects of reduced retinal blood flow on cone and rod cells.

## Data availability statement

The raw data supporting the conclusions of this article will be made available by the authors, without undue reservation.

## Ethics statement

The studies involving humans were approved by Institutional Review Board (or Ethics Committee) of Biomedical Ethics Committee of Beihang University. The studies were conducted in accordance with the local legislation and institutional requirements. The participants provided their written informed consent to participate in this study.

## Author contributions

YW: Investigation, Methodology, Project administration, Software, Writing – review & editing. JX: Writing – original draft, Data curation, Formal analysis, Investigation, Writing – review & editing. XY: Software, Writing – review & editing, Investigation, Methodology, Project administration. YL: Writing – review & editing, Investigation, Methodology, Project administration, Software. ZW: Writing – review & editing, Data curation, Investigation. AG: Writing – review & editing, Data curation, Investigation. YD: Writing – review & editing, Data curation, Investigation. XZ: Writing – review & editing, Data curation, Investigation. SL: Writing – review & editing. JL: Writing – review & editing, Data curation, Investigation. CZ: Writing – review & editing, Data curation, Investigation. YL: Writing – review & editing, Data curation, Investigation. ZL: Writing – review & editing, Project administration, Supervision, Validation. XL: Writing – review & editing, Conceptualization, Data curation, Formal analysis, Funding acquisition, Investigation, Methodology, Project administration, Resources, Software, Supervision, Validation, Visualization. LD: Writing – review & editing, Conceptualization, Data curation, Formal analysis, Funding acquisition, Investigation, Methodology, Project administration, Resources, Software, Supervision, Validation, Visualization.

## References

[B1] Akberova S. I. Markitantova Y. V. Ryabtseva A. A. Stroeva O. G. (2016). Hypoxia as pathogenic factor affecting the eye tissues: the selective apoptotic damage of the conjunctiva and anterior epithelium of the cornea. Dokl. Biochem. Biophys. 467, 150–152. 10.1134/S1607672916020198 27193721

[B2] Benedek K. Kéri S. Grósz A. Tótka Z. Tóth E. Benedek G. (2002). Short-term hypobaric hypoxia enhances visual contrast sensitivity. Neuroreport 13, 1063–1066. 10.1097/00001756-200206120-00017 12060809

[B3] Brennan L. Disatham J. Kantorow M. (2020). Hypoxia regulates the degradation of non-nuclear organelles during lens differentiation through activation of HIF1a. Exp. Eye Res. 198:108129. 10.1016/j.exer.2020.108129 32628953 PMC7508769

[B4] Connolly D. M. (2011). Oxygenation state and twilight vision at 2438 m. Aviat. Space Environ. Med. 82, 2–8. 10.3357/ASEM.2904.2011 21235098

[B5] Connolly D. M. Hosking S. L. (2009). Oxygenation state and mesopic sensitivity to dynamic contrast stimuli. Optom. Vis. Sci. 86, 1368–1375. 10.1097/OPX.0b013e3181be9d89 19797993

[B6] Davis C. Hackett P. (2017). Advances in the Prevention and Treatment of High Altitude Illness. Emerg. Med. Clin. North Am. 35, 241–260. 10.1016/j.emc.2017.01.002 28411926

[B7] dell'Omo R. Semeraro F. Bamonte G. Cifariello F. Romano M. R. Costagliola C. (2013). Vitreous mediators in retinal hypoxic diseases. Mediators Inflamm. 2013:935301. 10.1155/2013/935301 23365490 PMC3556845

[B8] Feigl B. Stewart I. Brown B. (2007). Experimental hypoxia in human eyes: implications for ischaemic disease. Clin. Neurophysiol. 118, 887–895. 10.1016/j.clinph.2006.12.012 17307390

[B9] Galletti C. Fattori P. (2018). The dorsal visual stream revisited: Stable circuits or dynamic pathways? Cortex. 98, 203–217. 10.1016/j.cortex.2017.01.009 28196647

[B10] Gekeler K. Schatz A. Fischer M. D. Schommer K. Boden K. Bartz-Schmidt K. U.. (2019). Decreased contrast sensitivity at high altitude. Br. J. Ophthalmol. 103, 1815–1819. 10.1136/bjophthalmol-2018-313260 30770358

[B11] Ishigaki H. Miyao M. (1993). Differences in dynamic visual acuity between athletes and nonathletes. Percept. Mot. Skills. 77, 835–839. 10.2466/pms.1993.77.3.835 8284163

[B12] Jiang J. Lei S. Zhu M. Li R. Yue J. Chen J.. (2021). Improving the generalizability of infantile cataracts detection via deep learning-based lens partition strategy and multicenter datasets. Front Med. 8:664023. 10.3389/fmed.2021.664023 34026791 PMC8137827

[B13] Krusche T. Limmer M. Jendrusch G. Platen P. (2020). Influence of natural hypobaric hypoxic conditions on dynamic visual performance. High Alt. Med. Biol. 21, 1–11. 10.1089/ham.2019.0033 31746645

[B14] Nassi J. J. Callaway E. M. (2009). Parallel processing strategies of the primate visual system. Nat. Rev. Neurosci. 10, 360–372. 10.1038/nrn2619 19352403 PMC2771435

[B15] Pang K. Lennikov A. Yang M. (2021). Hypoxia adaptation in the cornea: current animal models and underlying mechanisms. Animal Model Exp Med. 4, 300–310. 10.1002/ame2.12192 34977481 PMC8690994

[B16] Panjwani U. Thakur L. Anand J. P. Malhotra A. S. Banerjee P. K. (2006). Effect of simulated ascent to 3500 meter on neuro-endocrine functions. Indian J. Physiol. Pharmacol. 50, 250−256. 17193896

[B17] Ramsey D. J. Arden G. B. (2015). Hypoxia and dark adaptation in diabetic retinopathy: interactions, consequences, and therapy. Curr. Diab. Rep. 15, 118. 10.1007/s11892-015-0686-2 26493191

[B18] Rosengarten B. Kaps M. (2010). A simultaneous EEG and transcranial Doppler technique to investigate the neurovascular coupling in the human visual cortex. Cerebrovasc. Dis. 29, 211–216. 10.1159/000267840 20029192

[B19] Schatz A. Willmann G. Fischer M. D. Schommer K. Messias A. Zrenner E.. (2013). Electroretinographic assessment of retinal function at high altitude. J. Appl. Physiol. 115, 365–372. 10.1152/japplphysiol.00245.2013 23722709

[B20] Shinojima A. Lee D. Tsubota K. Negishi K. Kurihara T. (2021). Retinal diseases regulated by hypoxia-basic and clinical perspectives: a comprehensive review. J. Clin. Med. 10:5496. 10.3390/jcm10235496 34884197 PMC8658588

[B21] Stepanek J. Pradhan G. N. Cocco D. Smith B. E. Bartlett J. Studer M.. (2014). Acute hypoxic hypoxia and isocapnic hypoxia effects on oculometric features. Aviat. Space Environ. Med. 85, 700–707. 10.3357/ASEM.3645.2014 25022157

[B22] VerMaas J. R. Gehringer J. E. Wilson T. W. Kurz M. J. (2019). Children with cerebral palsy display altered neural oscillations within the visual MT/V5 cortices. Neuroimage Clin. 23:101876. 10.1016/j.nicl.2019.101876 31176292 PMC6555897

[B23] Wist E. R. Ehrenstein W. H. Schrauf M. (1998). A computer-assisted test for the electrophysiological and psychophysical measurement of dynamic visual function based on motion contrast. J. Neurosci. Methods. 80, 41–47. 10.1016/S0165-0270(97)00196-9 9606049

